# Are Exercise-Induced Premature Ventricular Contractions Associated with Significant Coronary Artery Disease?

**DOI:** 10.3390/jcm13226735

**Published:** 2024-11-08

**Authors:** Sok-Sithikun Bun, Clementine Massimelli, Didier Scarlatti, Fabien Squara, Emile Ferrari

**Affiliations:** Faculty of Medicine, Pasteur University Hospital, 06000 Nice, France; massimelli.c@chu-nice.fr (C.M.); scarlatti.d@chu-nice.fr (D.S.); ferrari.e@chu-nice.fr (E.F.)

**Keywords:** exercise-induced premature ventricular contractions, exercise stress testing, coronary artery disease

## Abstract

**Objectives:** Exercise-induced premature ventricular contractions (EIPVC) have been associated with higher mortality, but the association with coronary artery disease (CAD) has not been precisely established. Our objective was to assess in a group of subjects with EIPVC and cardiovascular risk factors the association with underlying significant coronary artery disease (CAD), in comparison with a control group of patients with cardiovascular risk factors and exercise test (ET) showing ischaemia. **Methods:** All the patients (above 35 years old) referred for ET at our institution were prospectively included. Patients with at least one cardiovascular risk factor and without known CAD were divided into 2 groups: group A if EIPVC were present (either during exercise or during recovery), at least more than 10% over 30 s of recording; group B if ET was showing ischaemia. The presence of CAD was then confirmed in both groups by coronary arteriography, and/or thallium scintigraphy, and/or cardiac MRI and/or coronary CT angiography performed within 2 months after ET realization. **Results:** From November 2020 to December 2022, 4098 ETs were performed. After exclusion (normal ETs = 2194; known CAD = 1109; age < 35 years old = 487; congenital heart disease = 59; mitral valve prolapse = 4), 46 patients with EIPVC were finally identified (male 65%, mean age 61.5 ± 11 years), and 71 in group B. CAD was confirmed using additional tests in 5/46 (11%) patients in group A versus 38/71 (54%) in group B (*p* < 0.0001). **Conclusions:** Amongst patients without known CAD, the presence of EIPVC was less frequently associated with an underlying CAD, compared to the presence of exercise-induced “electrical” ischaemia.

## 1. Introduction

Premature ventricular contractions (PVC) have been reported to predict heart failure and death in subjects without preexisting structural heart disease [1]. Exercise stress testing (ET) may be proposed in the diagnosis of underlying coronary artery disease (CAD) if imaging tests are not available [2]. Despite lower sensitivity and specificity, ET is still the first line exam to be proposed by the French Society of Cardiology for evaluating the presence of CAD in patients with low to intermediate pretest probability [3]. Exercise-induced premature ventricular contractions (EIPVC) may occur during ET in asymptomatic patients in a frequency ranging from 3.7 to 27% according to previous studies [4,5]. EIPVCs in asymptomatic middle-aged men have been associated with higher mortality during longitudinal follow-up [6]. Other studies showed that PVCs during the recovery phase were more predictive of an increased risk of death, in comparison with the exercise phase [7,8]. The exact mechanism of this association remains unclear. Some authors reported that PVC during the recovery phase of a stress test predicted myocardial ischaemia on myocardial perfusion imaging [9]. Nevertheless, in this study, more than one third of the patients already had a history of CAD. The association of EIPVC with concomitant myocardial ischaemia in subjects without previously known CAD has yet to be confirmed.

Our objective was to assess in a group of subjects with EIPVC and cardiovascular risk factors (without previously known CAD) the association with underlying significant CAD, in comparison with a control group of patients with cardiovascular risk factors and ET showing ischaemia.

## 2. Materials and Methods

### 2.1. Patient Population

From November 2020 to December 2022, all the subjects (above 35 years old) referred for ET at our institution (Pasteur University Hospital, Nice, France) were prospectively included. The present study was a prospective non-randomized case-control study in a single secondary centre. Individuals with cardiovascular risk factors (≥1) and without previously known CAD were divided into 2 groups after ET: group A if EIPVC were present (either during exercise or during recovery), at least more than 10% over 30 s of recording; group B if ET revealed ischaemic response. Considered cardiovascular risk factors were as follow: Age > 55 in male or >65 in female patients, family history (major cardiovascular event and/or sudden cardiac death occurring under the age of 55 in a male relative/under the age of 65 in a female relative), diabetes, hyperlipidaemia with treatment, hypertension, and tobacco use.

### 2.2. Exercise Test Protocol

Bicycle ETs were performed by the same operator in both groups, using standard Bruce protocol. It consisted of successive workloads, increasing from 30 W every 2 min. Cardiac rhythm was continuously monitored, and 30 s recordings were made from a bipolar lead (V5 and V5R) while the subject was at rest and every 2 min, at maximal effort, and every 1 min during the 4 min recovery time, or whenever the monitoring physician observed an arrhythmia. ET was terminated if the subject had fatigue, dyspnoea, leg discomfort, any chest pain, systolic blood pressure above 250 mm Hg, sustained ventricular tachycardia, or ECG changes indicative of ischaemia. At the time of each ECG recording, the frequency of PVC was assessed as the maximal number of premature ventricular depolarizations divided by the total number of ventricular depolarizations during any of the 30 s recordings. Subjects with a run of two or more consecutive PVC or with PVC constituting more than 10 percent of all ventricular depolarizations on any of the ECGs recorded with the subject during exercise, or during recovery were classified as having had frequent PVC during that period. An ischaemic response was considered in case of J-point depression >1.0 mm with a flat or downsloping ST-segment in both precordial and standard leads, for at least 30 s during the peak of exercise or during the recovery from exercise.

### 2.3. CAD Tests

The presence of CAD was then confirmed (at the discretion of the referring cardiologist) in both groups by thallium scintigraphy, and/or cardiac magnetic resonance (CMR) imaging, and/or computed tomography (CT) angiography, and/or eventually completed by coronary arteriography performed within 2 months after ET realization. A positive tomogram was defined by a segmental perfusion defect on the immediate postexercise images in ≥ two contiguous slices and two image orientations during thallium scintigraphy. The presence of obstructive disease was confirmed as detection of at least one ≥50% coronary artery luminal diameter stenosis by CT, and/or ≥70% coronary artery luminal diameter stenosis during the angiogram. The patients were excluded from analysis if CAD tests were not performed within the two-month period after ET realization.

In their cardiac work-up, patients from group A were asked to undergo 24 h Holter recordings at the discretion of the referring cardiologist. This study was approved by the Institutional Review Board. 

### 2.4. Statistical Analysis

The statistical analysis was performed using Excel (San Diego, CA, USA). Categorical variables were described as numbers and percentages. Continuous variables were described as mean ± SD for variables with normal distributions or as median with range for variables not normally distributed. Between groups, differences of categorical variables were compared using chi-squared test. Differences between continuous variables were compared using a student *t*-test. Considering 20% of myocardial ischaemia in patients with EIPVC from a previous study using SPECT imaging [10], with a power of 80% and an α error rate at 0.05 (two-sided test), the sample size is 43/group, 86 in total (sample size for ANCOVA, Nquery v 9.1). 

## 3. Results

**Figure 1:**
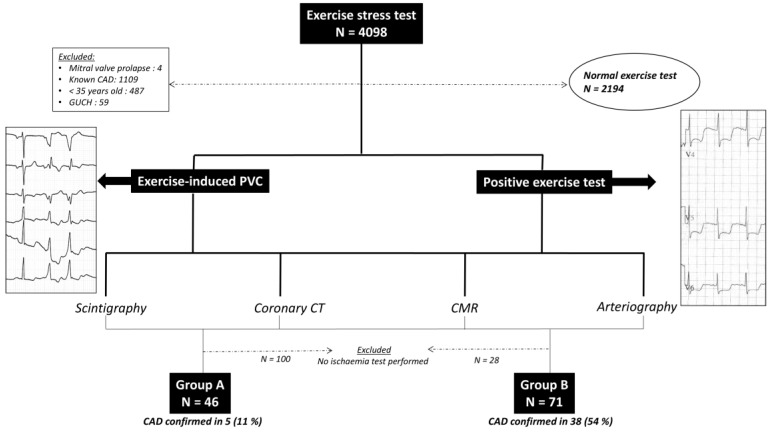
Flow chart of patients inclusion and exclusion.

A total of 4098 ETs were performed in our institution during this study period (Figure 1). After exclusion (normal ETs = 2194; known CAD = 1109; age < 35 years old = 487; congenital heart disease = 59; mitral valve prolapse = 4; patients refusing additional tests for ischaemia = 128), 46 patients with EIPVC were finally identified (male 65%, mean age 61.5 ± 10.8 years), and 71 in group B (male 69%, mean age 61.7 ± 10.1 years) (Table 1). The indications for ET realization in group A were: CAD detection (n = 19, 41%); PVC work-up (n = 7, 15%); preoperative cardiac assessment (n = 4, 10%); palpitations (n = 4, 10%); short breathing symptom (n = 3, 6%); competitive sports screening (n = 4, 10%); syncope during effort (n = 3, 6%); and atypical chest pain (n = 2, 4%). ET was performed in group B for: CAD detection (n = 36, 50%); preoperative cardiac assessment (n = 13, 18%); atypical chest pain (n = 9, 12%); short breathing symptom (n = 3, 4%); aortic valve stenosis (n = 3, 4%); syncope during effort (n = 3, 4%); and other (n = 6, 8%). The groups were comparable concerning cardiovascular risk factors (except concerning smoking and family history), without any significant difference concerning mean left ventricular ejection fraction (61 ± 9.3% versus 63 ± 7%, *p* = 0.27). Only 3 patients (4.2%) from group B presented with EIPVC > 10% over a 30 s period. EIPVC occurred in the exercise phase in 31/46 (67%) of the patients, during the recovery phase in 22/46 (48%) of the patients, and both in 17/46 (37%) of the patients. Twenty-four h Holter ECG was performed in 39/46 (85%) patients in group A. The mean number of PVCs on 24 h Holter recordings (group A) was 5773 ± 8290. The PVCs were from left origin in 23/46 (50%), right origin in 22/46 (48%), and arising from both ventricles in 1/46 (2%). No patient elicited PVCs with bidirectional morphology; PVCs were monomorphic in 41/46 (89.2%) patients, and polymorphic in 5/46 (10.8%). ST-segment depression suggesting ischaemia was observed in 4/46 (8.6%) of the patients from group A. After the full cardiac work-up (as shown in Table 2), and after exclusion of patients who did not undergo CAD tests within 2 months after ET, CAD was finally confirmed in 5/46 (11%) patients in group A versus 38/71 (54%) in group B (*p* = 0.0001). Clinical characteristics of the patients in whom CAD has been confirmed in both groups are available as Supplementary Material.

## 4. Discussion

The main finding of our current prospective study is that underlying CAD was detected in 11% of patients presenting with EIPVC (exercise or recovery phase) in contrast with 54% in case of positive ET. In comparison with previously published studies, the presence of CAD was confirmed in our study by modern tests (scintigraphy, CMR or arteriography), and not limited to myocardial ischaemia confirmation by stress ECG only. 

Another finding concerns the low prevalence of EIPVC (1.1%) in a population of subjects or patients referred for ET in a medium volume centre [2]. This is in agreement with previous studies reported in the literature. Of note, the prevalence of positive ET represented 1.7% of our initial population of subjects/patients, which is a rare situation in daily practice. ET may be routinely proposed for diagnosis and risk stratification of symptomatic patients with known and suspected CAD. Additionally, ET may be used for risk assessment of apparently healthy persons, or intermediate-risk asymptomatic adults before starting exercise programs, or patients at high risk for CAD because of comorbidities [11,12].

### 4.1. Prognostic Value of EIPVCs in the Literature

The prognostic value of EIPVCs has been previously studied in asymptomatic cohorts and in patients referred for ET. Jouven et al. published a cohort of 6.101 asymptomatic middle-aged men who performed standardized graded ET. Among them, 138 (2.3%) elicited EIPVC during exercise (>2 consecutive PVCs or PVCs comprising > 10% of heart beats during any 30-s period), and had significantly higher risk of cardiovascular mortality (CVM) (risk ratio [RR]: 2.53; 95% CI: 1.65–3.88; *p* < 0.001) over a long-term follow-up of 23 years, similar to that associated with ischaemic changes on stress ECG (RR: 2.63; 95% CI: 1.93–3.59; *p* < 0.001) [6]. In this study, EIPVCs during recovery were observed in 174 (2.9%) individuals with a higher rate of non-cardiovascular death. Interestingly, only 6% of the 138 subjects with EIPVCs during exercise also had stress ECG suggestive for ischaemia during ET. The authors concluded that EIPVCs were not related to the presence of exercise-induced myocardial ischaemia, because subjects with a positive ET suggestive of ischaemia and subjects with EIPVCs did not have the same pattern of risk factors (lower BMI and higher tobacco consumption in this group) [13]. This is a major difference with our mixed population (35% female), which is a real-life cohort of subjects referred for ET in a cardiology department with a mean age of 61.5 ± 10.8 years versus men from 42 to 53 years only in the study by Jouven et al. To be included in our study, subjects should have at least one cardiovascular risk factor. We hypothesized that the prevalence of underlying myocardial ischaemia would be different in an older population with cardiovascular risk factors and EIPVC. Actually, myocardial ischaemia was confirmed by stress ECG only during ET in previous studies, while stress imaging (including invasive) tests were required to confirm the presence of underlying myocardial ischaemia in our cohort, raising a prevalence of 11% of CAD. More recently, Refaat et al. showed in a retrospective cohort of 5.486 asymptomatic subjects (mean baseline age 45.4 ± 10.8 years, 42% women) that high-grade EIPVCs during recovery (2.4% of the population) were associated with higher CVM (hazard ratio [HR]: 1.68; 95% CI: 1.19–2.79; *p =* 0.006), while high-grade PVCs during exercise (occurring in 1.8% of the individuals) were not during a mean follow-up of 20.2 years [8]. The authors hypothesized that PVCs during recovery would be related to the presence of pathologic myocardial substrates. Previous studies showed conflicting results concerning the prognostic value of EIPVCs during exercise or recovery. Mora et al. showed in a population of 2.994 North American women (aged 30 to 80 years) that EIPVCs were associated with higher CVM (HR: 1.69; 95% CI: 1.11–2.58; *p* = 0.02), but not all-cause mortality (ACM) over 20 years of follow-up [14]. In a previous meta-analysis of ten observations, Lee et al. reported that EIPVCs were associated with a pooled RR of 1.82 (95% CI: 1.84–2.30) of developing cardiovascular events (including non-fatal myocardial infarction and angina), CVM and ACM over a mean follow-up of 16 years [15]. A subgroup analysis from Kim et al. showed that EIPVCs during recovery were significantly associated with a higher risk of ACM [16]. The latest meta-analysis pooling 13 studies including 82.161 individuals (mean age 49.3 years) confirmed that EIPVCs (only frequent) were associated with both elevated risk of ACM (RR: 1.30; 95% CI: 1.18–1.42; *p* < 0.001) and CVM (RR: 1.67; 95% CI: 1.40–1.99; *p* < 0.001). The authors found that PVCs during the recovery phase in predicting the interest outcomes was superior compared to the PVCs occurring during the exercise phase [17].

Lindow et al. suggested in their large cohort study that PVCs were only associated with a higher risk of death when structural heart dysfunctions were present, suggesting that baseline echocardiography may be necessary [18]. Of note, patients with PVCs during the recovery phase had a negligible difference in mean LVEF, compared to those without PVCs. Mean LVEF was not statistically different between the two groups in our study, but the control group included patients with positive ET. Actually, in the study by Morshedi-Meibodi et al., echocardiographic abnormalities were not related to an increased risk of mortality in their adjusted-model analysis [4]. 

### 4.2. Prevalence of CAD in Intermediate-Risk Population

The estimated prevalence of angina ranges from 2–5% in men aged 45–54 to 10–20% in men aged 65–74 [19]. The sensitivity of ET for detecting significant CAD ranges between 23 and 100% (mean 68%) [20]. Given superior performances (sensitivity) of additional imaging techniques, they were performed in our study in both groups. Our reported sensitivity in a cohort of 71 patients in group B (mean age 61.7 ± 10.1 years, 69% male, with at least one cardiovascular risk factor) was 54%, the diagnostic yield having been increased by imaging tests and/or invasive arteriography. In patients with known CAD, frequent PVCs (>0.6 PVC/h) following myocardial infarction were an independent predictor of CAD severity [21]. In apparently healthy subjects, an early study reported that «abnormal» PVCs recorded on a 24 h Holter monitoring were strongly associated with subsequent CAD [22]. Nevertheless, the study included a limited group of six patients developing CAD out of thirteen with frequent PVCs. Community based studies showed that the presence of PVCs is correlated to a 1.2-fold increased risk for the development of CAD over a 10 year follow-up period [23,24,25,26]. Considering EIPVCs, the association with ischaemia has been and still is a subject of debate [9]. Recently, using SPECT, myocardial ischaemia was found in almost 30% of the patients with EIPVCs from right bundle morphology, and 19.4% in patients with left bundle EIPVCs [10]. This prevalence is two-fold higher than our EIPVCs group, but our cohort excluded patients with known CAD, while representing one third of the population in the study by Biere et al. [10]. The originality of our studied population was to exclude patients with a history of CAD. 

Finally, if myocardial ischaemia may not represent the only substrate for EIPVCs, their negative prognostic outcome (ACM, CVM, including myocardial infarction) may be explained by autonomic dysregulation [8]. It has been reported that vagal impairment can occur in individuals during the recovery phase, creating a pro-arrhythmic substrate inducing PVCs [27]. The authors suggested that parasympathetic dysfunction as manifested by attenuation of heart rate recovery was correlated with an increased risk of ACM, regardless of the presence of PVCs. More recently, some authors reported the association of concave-shaped chest wall (as measured by the modified Haller Index) with EIPVC. The suggested mechanism was a possible compression on the cardiac chambers [28]. Interestingly, the authors showed that these patients had a good prognosis during the mid-term follow-up period. It would be interesting to measure this morphological index in patients presenting with EIPVC. 

### 4.3. Clinical Implications

Underlying CAD was reported in 11% of patients with EIPVC in our study. Given the negative long-term prognosis of EIPVC (especially during recovery), a regular cardiological follow-up is recommended in individuals with EIPVC. Nevertheless, EIPVCs per se do not justify the realization of additional imaging and/or invasive tests in asymptomatic individuals, even with cardiovascular risk factors, which should be symptom-driven, independently from the presence of EIPVC. 

### 4.4. Limitations

Our study included a limited number of patients, but the study was not designed to assess the longitudinal follow-up of our cohort. The sensitivity for detecting significant CAD in asymptomatic patients was increased using stress imaging techniques—at the discretion of the referring cardiologist—which were not equally distributed between the two groups. Three different imaging techniques have been performed in our population (after ET realization), with variable respective sensitivity and specificity, which may represent another limitation. Because of a strict deadline and methodology restriction (within two months after ET realization), some patients with scheduled additional exams were excluded if performed after the two month period after ET. A large number of patients refused to undergo additional imaging and/or functional tests after ET, which represents another main limitation in our study, but this reflects the real-life situation. Coronary arteriography was significantly less performed in group A, which may have led to the underestimation of CAD in subjects with EIPVC. One may hypothesize that a higher prevalence of CAD would have been found in group A if more angiographies were to be performed in this group. Similarly, only few CMRs were performed in group B, while the half of group A underwent CMR evaluation. This seems clinically relevant as CMR is usually recommended in the diagnostic work-up of patients with EIPVCs [29]. Some patients could not have a 24 h ECG Holter monitoring (15%). Noteworthily, PVCs burden was not assessed in previously published studies concerning EIPVCs.

## 5. Conclusions

Among patients with at least one cardiovascular risk factor, but without known CAD prior to the ET, the presence of EIPVC (either during effort or recovery) was less frequently associated with an underlying CAD, compared to the presence of exercise-induced “electrical” ischaemia. The presence of EIPVCs per se in asymptomatic patients may not justify the need for invasive coronary evaluation. Given their negative prognostic value, occurrence of EIPVC, especially during the recovery phase, (moreover in subjects of cardiovascular risk factors) should push physicians to propose a regular cardiologic follow-up in these subjects. 

To fully understand the long-term implications of EIPVCs, a longitudinal follow-up study is warranted. This larger multicentre study would allow for the assessment of CAD development, patient outcomes, and the potential need for interventions in patients with EIPVCs.

## Figures and Tables

**Table 1:** Characteristics of patients.

	EIPVC Group A (n = 46)	Abnormal Exercise Test Group B (n = 71)	*p*-Value
Age (y)	61.5 ± 10.8	61.7 ± 10.1	0.93
Male, n (%)	30 (65)	49 (69)	0.67
BMI (kg/m^2^)	26.5 ± 6.3	24.8 ± 3.6	0.10
Hyperlipidaemia, n (%)	9 (20)	26 (37)	0.049
Diabetes, n (%)	7 (15)	15 (21)	0.42
Hypertension, n (%)	20 (43)	24 (34)	0.29
Family history, n (%)	6 (13)	21 (30)	0.038
Tobacco use, n (%)	17 (37)	13 (18)	0.024
Mean LVEF (%)	61 ± 9.3	63 ± 7.0	0.27

LVEF: Left ventricular ejection fraction.

**Table 2:** Significant coronary artery disease detection in both groups.

	EIPVC Group A (n = 46)	Abnormal Exercise Test Group B (n = 71)	*p*-Value
Mean ST depression (mm)	0.89 ± 0.6	1.9 ± 0.65	<0.0001
Stress echocardiography, n (%)	0 (0)	1 (1)	0.42
Positive	0 (0)	0 (0)	1
CT angiography, n (%)	8 (17)	14 (20)	0.75
Positive	0 (0)	7 (10)	0.028
Thallium scintigraphy, n (%)	7 (15)	8 (11)	0.53
Positive	1 (2)	2 (3)	0.83
CMR, n (%)	23 (50)	5 (7)	<0.0001
Positive	1 (2)	1 (1)	0.76
Arteriography, n (%)	19 (41)	50 (70)	0.0018
One vessel lesions	1 (2)	16 (23)	0.0023
Two vessels lesions	2 (4)	15 (21)	0.012
Three vessels lesions	2 (4)	7 (10)	0.27
CAD confirmed, n (%)	5 (11)	38 (54)	<0.0001

## Data Availability

The data presented in this study are available on request from the corresponding author. The data are not publicly available due to privacy.

## Supplementary Materials

The following supporting information can be downloaded at: https://www.mdpi.com/article/10.3390/jcm13226735/s1, Table S1: Characteristics of patients with coronary artery disease confirmation in both groups.

## References

[1] Dukes J.W., Dewland T.A., Vittinghoff E., Mandyam M.C., Heckbert S.R., Siscovick D.S., Stein P.K., Psaty B.M., Sotoodehnia N., Gottdiener J.S. (2015). Ventricular Ectopy as a Predictor of Heart Failure and Death. J. Am. Coll. Cardiol..

[2] Knuuti J., Wijns W., Saraste A., Capodanno D., Barbato E., Funck-Brentano C., Prescott E., Storey R.F., Deaton C., Cuisset T. (2020). 2019 ESC Guidelines for the diagnosis and management of chronic coronary syndromes. Eur. Heart J..

[3] Marcadet D.M. (2019). Nouvelles recommandations concernant la pratique des tests d’effort en cardiologie [Exercise testing: New guidelines]. Presse Med..

[4] Morshedi-Meibodi A., Evans J.C., Levy D., Larson M., Vasan R. (2004). Clinical correlates and prognostic significance of exercise-induced ventricular premature beats in the community: The Framingham Heart Study. Circulation.

[5] Marine J.E., Shetty V., Chow G.V., Wright J.G., Gerstenblith G., Najjar S.S., Lakatta E.G., Fleg J.L. (2013). Prevalence and prognostic significance of exercise-induced nonsustained ventricular tachycardia in asymptomatic volunteers: BLSA (Baltimore Longitudinal Study of Aging). J. Am. Coll. Cardiol..

[6] Jouven X., Zureik M., Desnos M., Courbon D., Ducimetière P. (2000). Long-term outcome in asymptomatic men with exercise-induced premature ventricular depolarizations. N. Engl. J. Med..

[7] Frolkis J.P., Pothier C.E., Blackstone E.H., Lauer M.S. (2003). Frequent ventricular ectopy after exercise as a predictor of death. N. Engl. J. Med..

[8] Refaat M.M., Gharios C., Moorthy M.V., Abdulhai F., Blumenthal R.S., Jaffa M.A., Mora S. (2021). Exercise-Induced Ventricular Ectopy and Cardiovascular Mortality in Asymptomatic Individuals. J. Am. Coll. Cardiol..

[9] Meine T.J., Patel M.R., Shaw L.K., Borges-Neto S. (2006). Relation of ventricular premature complexes during recovery from a myocardial perfusion exercise stress test to myocardial ischemia. Am. J. Cardiol..

[10] Bière L., Mezdad T.H., Dupuis J.M., Vervueren L., Rakotonirina H., Prunier F., Furber A. (2018). Long-term prognostic significance of right bundle-branch morphology ventricular ectopy induced during stress test in patients with intermediate to high probability of coronary artery disease. Europace.

[11] Greenland P., Alpert J.S., Beller G.A., Benjamin E.J., Budoff M.J., Fayad Z.A., Foster E., Hlatky M.A., Hodgson J.M., Kushner F.G. (2010). 2010 ACCF/AHA guideline for assessment of cardiovascular risk in asymptomatic adults: A report of the American College of Cardiology Foundation/American Heart Association Task Force on Practice Guidelines. Circulation.

[12] Wolk M.J., Bailey S.R., Doherty J.U., Douglas P.S., Hendel R.C., Kramer C.M., Min J.K., Patel M.R., Rosenbaum L., Shaw L.J. (2014). ACCF/AHA/ASE/ASNC/HFSA/HRS/SCAI/SCCT/SCMR/STS 2013 multimodality appropriate use criteria for the detection and risk assessment of stable ischemic heart disease: A report of the American College of Cardiology Foundation Appropriate Use Criteria Task Force, American Heart Association, American Society of Echocardiography, American Society of Nuclear Cardiology, Heart Failure Society of America, Heart Rhythm Society, Society for Cardiovascular Angiography and Interventions, Society of Cardiovascular Computed Tomography, Society for Cardiovascular Magnetic Resonance, and Society of Thoracic Surgeons. J. Am. Coll. Cardiol..

[13] Jouven X., Ducimetière P. (2001). Exercise testing: Do frequent premature ventricular depolarizations represent a new criterion of positivity?. Eur. Heart J..

[14] Mora S., Redberg R.F., Cui Y., Whiteman M.K., Flaws J.A., Sharrett A.R., Blumenthal R.S. (2003). Ability of exercise testing to predict cardiovascular and all-cause death in asymptomatic women: A 20-year follow-up of the lipid research clinics prevalence study. JAMA.

[15] Lee V., Perera D., Lambiase P. (2017). Prognostic significance of exercise-induced premature ventricular complexes: A systematic review and meta-analysis of observational studies. Heart Asia.

[16] Kim J., Kwon M., Chang J., Harris D., Gerson M.C., Hwang S.-S., Oh S.-W. (2016). Meta-Analysis of Prognostic Implications of Exercise-Induced Ventricular Premature Complexes in the General Population. Am. J. Cardiol..

[17] Iqbal M., Putra I.C.S., Kamarullah W., Pranata R., Achmad C., Karwiky G., Pramudyo M., Goenawan H., Akbar M.R., Kartasasmita A.S. (2022). Revisiting exercise-induced premature ventricular complexes as a prognostic factor for mortality in asymptomatic patients: A systematic review and meta-analysis. Front. Cardiovasc. Med..

[18] Lindow T., Ekström M., Brudin L., Hedman K., Ugander M. (2022). Prognostic implications of structural heart disease and premature ventricular contractions in recovery of exercise. Sci. Rep..

[19] Fox K., Garcia M.A., Ardissino D., Buszman P., Camici P.G., Crea F., Daly C., De Backer G., Hjemdahl P., Lopez-Sendon J. (2006). Guidelines on the management of stable angina pectoris: Executive summary: The Task Force on the Management of Stable Angina Pectoris of the European Society of Cardiology. Eur. Heart J..

[20] Gianrossi R., Detrano R., Mulvihill D., Lehmann K., Dubach P., Colombo A., McArthur D., Froelicher V. (1989). Exercise-induced ST depression in the diagnosis of coronary artery disease. A meta-analysis. Circulation.

[21] Minisi A.J., Mukharji J., Rehr R.B., Lewis S., Richardson D.W., Romhilt D.W., Vetrovec G.W. (1988). Association between extent of coronary artery disease and ventricular premature beat frequency after myocardial infarction. Am. Heart J..

[22] Bjerregaard P., Sorensen K.E., Molgaard H. (1991). Predictive value of ventricular premature beats for subsequent ischaemic heart disease in apparently healthy subjects. Eur. Heart J..

[23] Rabkin S.W., Mathewson F.A., Tate R.B. (1981). Relationship of ventricular ectopy in men without apparent heart disease to occurrence of ischemic heart disease and sudden death. Am. Heart J..

[24] Sajadieh A., Nielsen O.W., Rasmussen V., Hein H.O., Frederiksen B.S., Davanlou M., Hansen J.F. (2006). Ventricular arrhythmias and risk of death and acute myocardial infarction in apparently healthy subjects of age >or=55 years. Am. J. Cardiol..

[25] Massing M.W., Simpson R.J., Rautaharju P.M., Schreiner P.J., Crow R., Heiss G. (2006). Usefulness of ventricular premature complexes to predict coronary heart disease events and mortality (from the Atherosclerosis Risk in Communities cohort). Am. J. Cardiol..

[26] Cheriyath P., He F., Peters I., Li X., Alagona P., Wu C., Pu M., Cascio W.E., Liao D. (2011). Relation of atrial and/or ventricular premature complexes on a two-minute rhythm strip to the risk of sudden cardiac death (the Atherosclerosis Risk in Communities [ARIC] study). Am. J. Cardiol..

[27] Qiu S., Cai X., Sun Z., Li L., Zuegel M., Steinacker J.M., Schumann U. (2017). Heart Rate Recovery and Risk of Cardiovascular Events and All-Cause Mortality: A Meta-Analysis of Prospective Cohort Studies. J. Am. Heart Assoc..

[28] Sonaglioni A., Rigamonti E., Nicolosi G.L., Lombardo M. (2021). Prognostic Value of Modified Haller Index in Patients with Suspected Coronary Artery Disease Referred for Exercise Stress Echocardiography. J. Cardiovasc. Echogr..

[29] Zeppenfeld K., Tfelt-Hansen J., de Riva M., Winkel B.G., Behr E.R., Blom N.A., Charron P., Corrado D., Dagres N., De Chillou C. (2022). 2022 ESC Guidelines for the management of patients with ventricular arrhythmias and the prevention of sudden cardiac death. Eur. Heart J..

