# Supplementary material for: Are Exercise-Induced Premature Ventricular Contractions Associated with Significant Coronary Artery Disease?
Source: J Clin Med. 2024 Nov 8;13(22):6735. doi: 10.3390/jcm13226735 (PMC11594783; doi:10.3390/jcm13226735)
Supplement: Supplementary file 1 [file jcm-13-06735-s001.zip › jcm-3282625-supplementary.pdf]

	Age / Gender	Indication for ET	Family history	Tobacco use	HTN	Diabetes	Hypercholesterolemia	BMI (kg.m²)	Scintigraphy (ischaemia)	Coronary CT/CAD confirmed	CMR /CAD confirmed	Arteriography	Stent(s) implantation
**Group A**													
1	56/M	Screening	0	1	1	0	0	19.9	1	1	1	3 vessels	Y
2	69/M	Screening	0	0	1	0	0	29.1	0	0	0	2 vessels	N
3	76/M	Screening	0	0	1	0	0	22.5	0	0	0	2 vessels	N
4	75/M	Preoperative	0	1	0	1	0	28.7	0	0	0	1 vessel	N
5	81/M	Short-breathing	0	0	1	0	1	28.4	0	0	0	3 vessels	N
**Group B**													
1	64/M	Pain	0	0	0	1	0	23.1	0	0	0	1 vessel	N
2	57/M	Pain	0	0	1	0	1	28.2	0	0	0	1 vessel	Y
3	58/M	Pain	1	0	0	0	0	27.0	0	0	0	3 vessels	N
4	75/M	Aortic stenosis	1	0	0	0	1	25.4	0	0	0	2 vessels	N
5	81/M	Aortic stenosis	0	1	1	0	0	33.0	0	0	0	2 vessels	N
6	64/M	Aortic stenosis	0	1	1	1	1	26.1	0	0	0	2 vessels	N
7	55/M	Screening	0	0	0	0	1	26.0	0	0	0	1 vessel	N

8	71/F	Screening	0	0	1	1	1	29.7	0	0	0	1 vessel	N
9	64/M	Screening	1	0	1	0	0	24.3	0	0	0	1 vessel	N
10	82/F	Screening	0	0	1	1	1	32.8	1	0	0	1 vessel	N
11	57/M	Screening	1	1	0	0	1	28.3	0	0	0	3 vessels	Y
12	61/M	Screening	1	1	0	0	1	27.8	0	0	0	3 vessels	Y
13	53/M	Screening	0	0	1	0	0	23.5	0	0	1	0	N
14	60/M	Screening	0	0	1	0	0	21.8	0	1 vessel	0	0	N
15	67/M	Screening	1	0	1	0	0	28.7	0	0	0	2 vessels	N
16	60/M	Screening	0	1	1	1	1	22.5	0	0	0	3 vessels	Y
17	85/M	Screening	0	0	1	0	1	22.2	0	0	0	2 vessels	Y
18	72/M	Screening	0	0	1	1	1	24.7	0	0	0	1 vessel	Y
19	60/M	Screening	1	0	0	0	0	20.5	1	0	0	0	N
20	72/M	Pain	0	0	1	1	1	29.7	0	0	0	3 vessels	N
21	52/M	Pain	1	0	0	0	0	25.2	0	2 vessels	0	0	N
22	40/M	Pain	1	0	0	0	0	24.6	0	1 vessel	0	0	N
22	72/M	Short-breathing	0	0	0	0	0	25.2	0	0	0	1 vessel	N
23	53/F	Short-breathing	1	0	0	0	0	29.2	0	0	0	1 vessel	N
24	61/M	Short-breathing	0	0	0	0	0	25.6	0	0	0	1 vessel	N

25	66/M	Preoperative	1	0	1	0	0	24.9	0	0	0	2 vessels	Y
26	68/M	Aortic stenosis	0	0	0	0	0	21.4	0	0	0	2 vessels	Y
27	85/M	Aortic stenosis	0	0	0	0	0	24.9	0	0	0	2 vessels	N
28	67/M	Screening	1	0	1	0	1	28.0	0	0	0	2 vessels	Y
29	77/M	Screening	0	0	0	0	1	24.0	0	1 vessel	0	1 vessel	N
30	59/F	Screening	0	0	0	0	1	26.3	0	0	0	1 vessel	Y
31	56/F	Screening	0	1	1	0	1	26.4	0	2 vessels	0	0	N
32	77/M	Screening	1	0	0	0	0	25.0	0	0	0	1 vessel	N
33	64/M	Screening	0	0	0	0	0	24.2	0	0	0	2 vessels	N
34	58/M	Screening	1	0	0	0	0	26.4	0	1 vessel	0	1 vessel	N
35	55/F	Screening	1	1	1	0	1	25.8	0	0	0	2 vessels	N
36	66/F	Screening	0	0	1	0	0	20.4	0	0	0	3 vessels	Y
37	73/M	Screening	0	0	0	1	1	32.2	0	0	0	2 vessels	Y
38	60/M	Syncope	0	0	0	0	0	24.7	0	2 vessels	0	2 vessels	N

BMI: body mass index; CAD: coronary artery disease; CMR: cardiac magnetic resonance imaging; CT: computed-tomography; ET: exercise test; HTN: hypertension.

Supplementary file Table S1. Characteristics of patients with coronary artery disease confirmation in both groups.
